# Injuries in male youth football: a one season prospective cohort study of 223 Danish elite players

**DOI:** 10.3389/fspor.2023.1250223

**Published:** 2023-12-18

**Authors:** Thomas Rostgaard Andersen, Andreas Drevsfeldt, Sören Möller, Merete Møller

**Affiliations:** ^1^ Department of Sports Science and Clinical Biomechanics, University of Southern Denmark, Odense, Denmark; ^2^ Danish Football Association, Brondby, Denmark; ^3^ Department of Clinical Research, University of Southern Denmark, Odense, Denmark; ^4^ Open Patient Data Explorative Network, Odense University Hospital, Odense, Denmark; ^5^ Research Unit for Musculoskeletal Function and Physiotherapy, Department of Sports Science and Clinical Biomechanics, University of Southern Denmark, Odense, Denmark; ^6^ Oslo Sports Trauma Research Center, Department of Sports Medicine, Norwegian School of Sports Sciences, Oslo, Norway

**Keywords:** player availability, prevalence, incidence, time-loss, injury burden, OSTRC-H2, health problems, soccer

## Abstract

**Objectives:**

This study prospectively investigated injury prevalence, incidence, and burden in male elite under-17 football players (*N *= 223*)* during a full season.

**Methods:**

The players weekly completed a standardized web-based injury survey (OSTRC-H2) and a physical exposure report throughout the study.

**Results:**

Average weekly response rate was 89.5%. Football exposure accounted for 52.4% of total physical exposure. On average (±SD), the players participated in individual football, strength, and rehabilitation practices for 1.2 ± 1.5, 3.0 ± .2.1, and 1.9 ± 3.4 h/week, respectively. In total, 742 health problems were reported. Mean weekly prevalence of health problems, injuries and illnesses were 20.1%, 16.5% and 3.8%, respectively. The injury incidence per 1,000 h of football exposure, match play and team practice were 8.28 (95% CI: 7.54–9.08), 16.77 (95% CI: 13.65–20.4), and 7.24 (95% CI: 6.5–8.04), respectively. Sudden-onset and gradual-onset injuries accounted for 36.7% and 43.4% of the total proportion of health problems. Hip/groin injuries had the highest incidence (1.58/1,000 h), whereas knee injuries had the highest burden (20.86 days lost/1,000 h). On average, the players experienced 3.33 health problems (average duration: 7.8 days). On average pr. player, 2.7 (95% CI: 2.2–3.3) wks of football exposure were lost.

**Conclusion:**

Sudden and gradual-onset injuries influenced player availability during the season. Health problem prevalence fluctuated markedly, and injury incidence was higher during match play than training. The players had substantial volumes of training beyond football-specific training and matches. Our findings could assist medical and sports science practitioneers in enhancing training and recovery processes to maximize player availability.

## Introduction

Despite the many health benefits of sports participation (1), it is also the leading cause of injury among children and adolescents (2). In a recent study involving 3,498 adults and 3,221 children, Danish senior and youth football players had together with handball the highest prevalence of injuries in the past 12 months compared to 49 other sports (3). Injuries sustained during youth may negatively impact player development, well-being, future performance, and career prospects (4, 5). Also, an increased risk of drop-out and osteoarthritis in later life has been reported (6, 7, 8). Preventing injuries and health problems in youth football is therefore of paramount importance.

An integral first step towards prevention is gaining an understanding of the extent of injuries and illnesses (9). Traditionally, sports injury surveillance research in football has focused on the identification of serious time-loss acute sudden-onset or medical attention injuries. In addition, injury severity has commonly been defined by the duration of time lost as a proxy measure using a medical staff, athletic trainers or non-medical staff for injury reporting (10, 11). Consequently, little is known about other injury types (e.g., non-traumatic injuries or those that do not result in time-loss or medical attention) as well as the consequences of injury besides time-loss.

Recent technological and methodological advances have resulted in new opportunities to measure sport-related injury using player self-reports (10, 12). OSTRC Overuse Injury Questionnaire was developed to identify the occurrence of overuse injuries and their consequences. This represents an important step in injury epidemiology as it identifies many injuries missed with traditional approaches.

The updated Oslo Sport Traumatic Research Center questionnaire on Health Problems (OSTRC-H2) serves as an injury surveillance method with a broader focus on health problems (13). This tool was recently employed to assess the efficacy of a load management intervention on injury risk in young elite football players (14), to prospectively monitor injury incidence within a single football academy (15), and to describe prevalence and burden of injuries and illnesses in Japanese men's university football players (16). Recent research indicates, that youth elite football players are at an increased risk of health problems during periods of rapid growth and maturation (17). Additional research suggest that the incidence of time-loss injuries and injury burden could be especially high in age groups below the age of 17 (18). However, to our knowledge, no studies have yet used the OSTRC-H2 method to examine the prevalence and burden of health problems in young elite football players who are under the age of 17 across multiple clubs. Conducting a large scale study could be especially important as previous studies suggest that the injury pattern in youth players, as opposed to elite adult players, may be characterized by a higher frequency of gradual-onset injuries (17).

Thus, the present study aimed to prospectively investigate the prevalence, incidence, and burden of health problems in a group of male youth elite football players competing in the Danish under-17 premier league over a full competitive season, utilizing the OSTRC-H2.

## Materials and methods

### Study design, recruitment, and ethical considerations

The study was conducted as a prospective one-season cohort study on male youth elite football players (age: 15–17 yrs.). The procedures in this study were parts of a comprehensive investigational protocol (The National Danish Male Youth Elite Football Study) initiated by The Danish League exploring injury pattern as well as changes to physical and psychological performance during a full competitive season in male youth football. The participating players were prior to the beginning of the study period selected for the 1st team player rooster of a Danish male under-17 premier league club by others. The players self-reported weekly health problems using the OSTRC-H2 (13) as well as training and match exposure during the 2021/22 in-season playing period including the mid-season break. Players were recruited through initial official contacts to the front office of all clubs competing in the male Danish under-17 premier league. Individual inclusion criteria were selected as follows: male youth elite football player included in the 1st team under-17 player rooster in a football club competing in the Danish under-17 premier league during the 2021/22 season. The exclusion criteria were selected as: not accepting participation in the study, and not being willing to adhere to all protocol procedures throughout the investigation period. Players provided written informed consent to participate in the study, with the informed consent form being signed by a parent/legal guardian. The study was reviewed and approved by the local ethic committee (Ethic Committee of Southern Denmark, Identifier: 20212000–89).

### Health problem and injury definitions

In accordance with the International Olympic Committee (IOC) consensus statement on method for recording and reporting epidemiological data in sports, and the football-specific extension of the IOC consensus statement (19, 20) a health problem was defined as: “*Any condition that reduced a player's normal state of complete health, irrespective of its consequences on football participation or performance, or whether the player sought medical attention”*. Health problems were classified into injuries and illnesses. An injury was defined as a tissue damage or other derangement of normal physical function. An illness was defined as a complaint or disorder experienced by a player, not related to injury. Injuries were further classified by mode of onset. A sudden-onset injury was defined as a single, clearly identifiable energy transfer. A gradual-onset injury was defined as multiple accumulative bouts of energy transfer without a single, clearly identifiable event being responsible for the injury (19). A time-loss related health problem was defined as a time-loss health problem leading to a player not being able to fully participate in a planned training session or match play (19). As per the football-specific extension of the IOC consensus statement, injury burden was defined as the number of days lost per 1,000 h of football exposure (20).

Football exposure was defined as the total hours of team training and matches, with team training exposure defined as the total number of hours of specific football training team practice. This included all team sessions involving the techniques and/or tactics of football. A match was defined as organized and scheduled match play against an opposing team (including official matches, friendlies and junior/reserve team matches and international matches) (19).

### Data collection

Player characteristics (age, height, weight and playing position) were collected at baseline prior to study start by club sports science sector staff members following standardized procedures. Player characteristics were subsequently reported to the study leaders.

**Figure 1:**
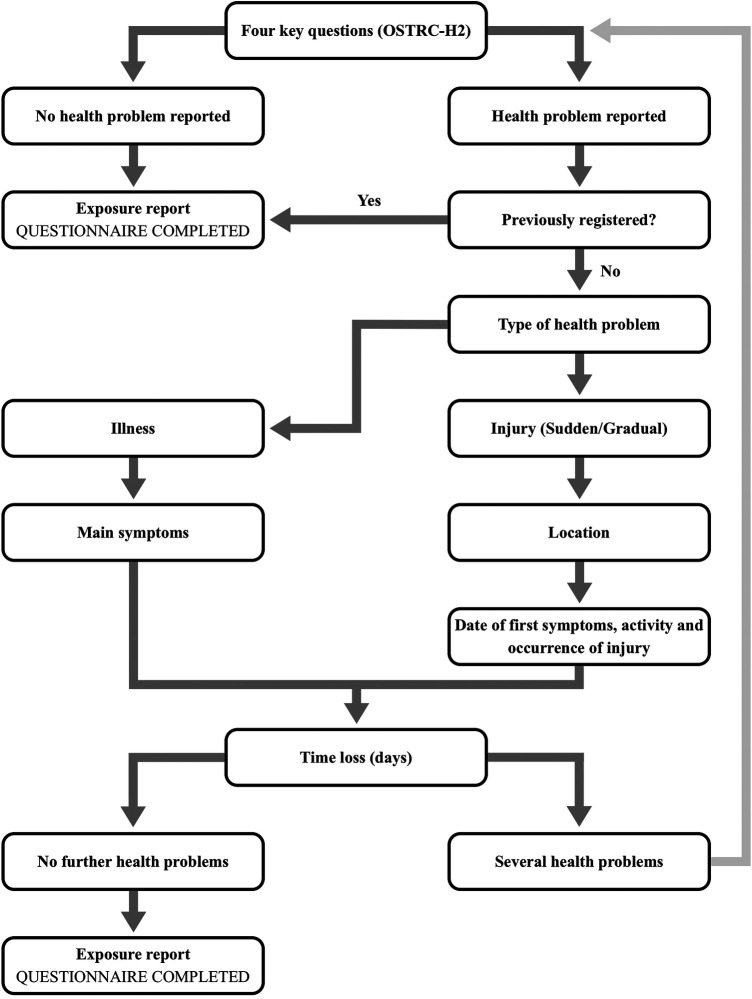
Weekly injury survey application logic. Diagram modified from Clarsen et al. (2013).

For a total of 45 weeks during the 2021/2022 season (August 2021 - June 2022), players reported any health problems experienced during the preceding 7 day period by completing the OSTRC-H2 (13) using a mobile application (AthleteMonitoring.com, FITSTATS Technologies Inc., Moncton, Canada) at the end of each week (Sunday at 7 p.m.). The standards and procedures of the OSTRC-H2 has previously been described (12). In brief, the OSTRC-H2 is based on four key questions (Figure 1) related to the difficulties in training and football participation (Q1, response options: 1 = full, 2 = full with a health problem, 3 = reduced with a health problem, 4 = absent due to a health problem), the extent to which participation was modified (Q2, response options: 1 = no modification, 2 = minor extent, 3 = moderate extent, 4 = major extent), the extent a health problem affected performance (Q3, response options: 1 = no effect, 2 = minor extent, 3 = moderate extent, 4 = major extent), and the extent of experienced symptoms/complaints (Q4, response options: 1 = no symptoms/complaints, 2 = mild extent, 3 = moderate extent, 4 = severe extent). Based on the responses to these questions, health problems were classified into any health problems (responses above 1 to Q1) or substantial health problems leading to a moderate or severe reduction in training volume or a moderate or severe reduction in sports performance (responses 3 or 4, respectively, for Q2 and Q3) (12). A time-loss injury was registered in the case players selected option 4 in Q1.

After completing the OSTRC-H2, the players reported their individual total weekly physical exposure over the last week by answering the following five question; 1) “*How many hours did you in total dedicate to team practice (not matches)?”*, 2) “*How many hours did you in total dedicate to self-practice and individual practice with the ball (not team practice)?”*, 3) “*How many hours did you in total dedicate to strength training including injury prevention?”*, 4) “*How many hours did you in total dedicate to self-practice and individual practice without the ball (not strength or injury prevention)?*”, 5) “*How many hours did you in total dedicate to rehabilitation from an injury or a health problem (all types of training)?”*.

The players were orally and in writing instructed on all technical matters related to the reporting process. At least one staff member at each club was assigned to support the players throughout the study period. To enhance individual response rates, the research team utilized a follow-up system in which individuals who did not complete the OSTRC-H2 or the individual exposure report on time received a text push-message. Also, a dedicated club staff member contacted them personally.

The team training and match exposure was cross-checked by evaluating micro-cycle training plans provided by the coaching staff, which were sent via e-mail to the study personnel in a pre-formatted Excel spreadsheet. Individual match exposure was confirmed by examining official match reports provided the Danish league association. All exposure values were reported weekly with the precision of 0.5 h, except match exposure which was reported with a precision of 1 min. Figure 1 illustrates the question logic of the weekly survey.

### Statistical analysis

The cumulative severity scores for all health problems were summed, with the proportion of the total burden of health problems made up by the number of illnesses and injuries (10). The weekly prevalence of health problems (e.g., injury and illness) was determined by dividing the number of players reporting any form of health problem by the number questionnaire respondents (12). The prevalence of substantial health problems was calculated using the same approach. The weekly prevalence of health problems and substantial health problems was reported as average percentages with 95% confidence intervals (95% CI). To assess player availability, the prevalence of each degree of participation was determined by calculation the number of players reporting each type of participation degree by the number of questionnaire respondents. The degree of participation was lead from OSTRC-H2 Q1, which divided the degree of participation into four categories: “*Full participation without any health problem”*, “*Full participation, but with a health problem”*, “*Reduced participation due to a health problem”,* or “*Could not participate due to a health problem”* (13). Injury incidence was expressed as the number of injuries per 1,000 h of both football team training and match play (football exposure) (21). Injury incidence was reported as mean values with 95% confidence intervals (95% CI). Mean injury burden was calculated as the number of injury days lost per 1,000 h (injury incidence multiplied by mean absence per injury divided by 1,000), accounting for both the frequency and severity of injuries (21). No attempts to impute missing health data were performed. Periods of vacation (e.g., winter break off-season period and periods during which the players had no planned training or matches) were considered a part of the total yearly macro-cycle and included for analysis (22). Duplicate OSTRC-H2 reports were excluded. All statistical analyses were conducted in Stata V.17.0 software (StataCorp, College station, TX, US).

## Results

### Participants

**Figure 2:**
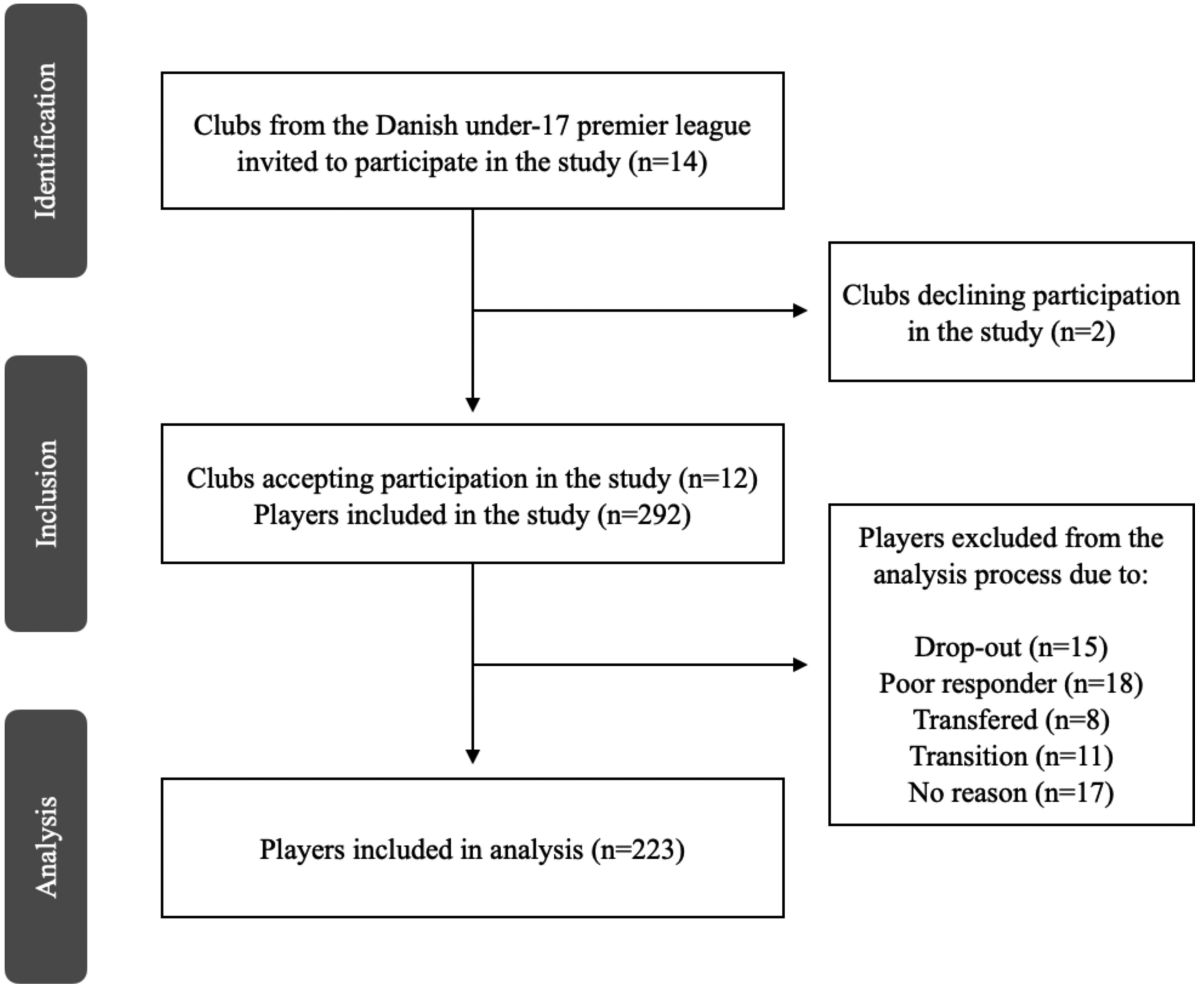
Recruitment and inclusion/exclusion flow chart of clubs and players from the Danish under-17 premier league included in the study.

Twelve out of fourteen eligible clubs agreed to participate in the study. In total, 292 players were individually invited to participate in the study, with 223 players included for final analysis (Figure 2). In total, 18 players were excluded due to an insufficient response rate, with the average response rate for the excluded players being 5.55 ± 0.0% (mean ± SD). During the study period, 8 players were additionally transferred to clubs outside the scope of present investigation, and 11 players were transferred to older age-group teams. These players were excluded from the final analysis. Furthermore, 32 players left the study within study period with reason (*N *= 15) or no reason (*N *= 17) provided. Baseline player characteristics are reported in Table 1. The mean weekly response rate during the study was 89.5% (95% CI 87.1 to 92.0).

**Table 1:** Player characteristics (*N* = 223).

Player characteristics, mean (SD)	
Age, years	15.6 ± 0.6
Height, cm	179.5 ± 6.4
Weight, kg	67.1 ± 7.8
BMI, kg/m^2^	20.7 ± 1.6
Playing position, *n* (%)	
Goalkeeper	21 (9.41)
Central defender	39 (17.49)
Full-back/Wing-back	46 (20.63)
Central midfielder	53 (23.77)
Wide midfielder/Wing	36 (16.14)
Striker/Central forward	28 (12.56)
Team, mean (SD)	
Size	19.3 ± 2.6

### Physical exposure

Total football exposure and training distribution are presented in Table 2. Football exposure accounted for more than half of the total yearly exposure time. Training activities conducted to increase strength qualities and reduce injury risk accounted for around 25% of total yearly exposure. Individual training with and without the ball and time dedicated to rehabilitations purposes accounted for approximately 25% of total yearly exposure.

**Table 2:** Weekly average and total exposure values per player during the 45-week study period (*N* = 223). Data are presented as mean ± SD. Yearly distribution is presented as percentage with 95% confidence intervals.

	Weekly average			Total			Yearly Distribution (%)	
Football exposure								
- Total football exposure (hours)	6.05	±	3.32	243.85	±	74.98	52.44	(50.53 to 54.35)
- Team training exposure (hours)	5.39	±	3.02	217.14	±	69.93	46.48	(44.80 to 48.16)
- Match play exposure (hours)	0.66	±	0.78	26.70	±	12.89	5.96	(5.53 to 6.38)
Football related exposure								
- Individual self-practice with ball exposure (hours)	1.18	±	1.54	47.47	±	33.95	9.66	(8.89 to 10.43)
Strength training and injury prevention exposure								
- Strength training and injury prevention exposure (hours)	2.99	±	2.1	120.39	±	56.87	24.48	(23.50 to 25.47)
Other activities								
- Rehabilitation and generic training activities (hours)	1.88	±	3.35	75.92	±	102.95	13.42	(11.72 to 15.12)

### Health problems

During the 45-week study period, a total of 742 health problems were reported. Based on severity score, injuries accounted for 80.2% of the total proportion of health problems (60.7% of all health problems). Sudden-onset and gradual-onset injuries accounting for 36.7% and 43.4% of the total proportion of health problems, respectively (28.0% and 32.7% of all cases, respectively). Lower extremity injuries accounted for 67.4% of the total proportion of injuries (equivalent to 51.6% of the total number of injuries). Illnesses accounted for 19.8% of the total proportion of health problems (39.2% of all reported health problems). Respiratory illness had the highest no. of illnesses (*N *= 126) (weekly average: 2.7 ± 2.6 cases), followed by COVID-19 (*N *= 82: weekly average: 1.8 ± 4.5 cases) (Table 3). On average, a player had 3.33 health problems throughout the study period, with an average duration of 7.8 days per health problem.

**Table 3:** Illnesses in danish male elite youth football players (*N* = 223) during the 45-week study period.

Categories	No. of illnesses	Weekly average
Cardiovascular	1	0.02 (0.15)
Gastrointestinal	23	0.49 (0.7)
Respiratory (upper and lower)	126	2.74 (2.66)
Dental	0	0.0 (0)
Neurological	6	0.12 (0.32)
Otological	1	0.02 (0.15)
Urogenital	0	0.0 (0)
Psychological	0	0.0 (0)
Dermatological	0	0.0 (0)
Ophthalmological	0	0.0 (0)
Nonspecific	21	0.44 (0.78)
Unknown	31	0.68 (0.82)
COVID-19	82	1.79 (4.54)
Musculoskeletal, rheumatological, and connective tissue (unrelated to injury).	0	0.0 (0)
Total	291	6.31 (5.69)

### Injury prevalence

**Figure 3:**
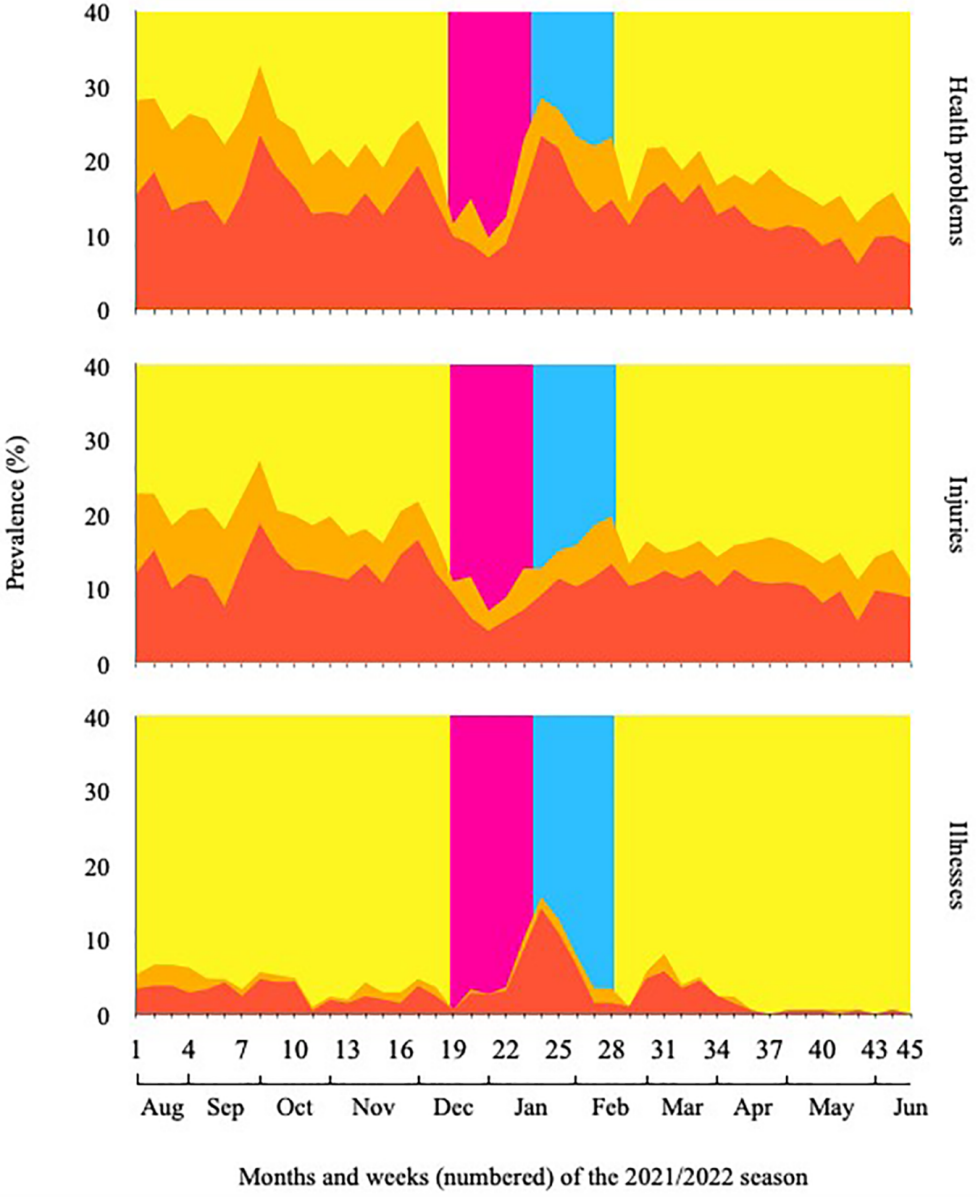
Weekly health problem (top), injury (mid), and illness (bottom) prevalence during the 45-week study period. Graph orange area: any complaint. Graph red area: substantial problem. Background color indication: Blue area = pre-season, Yellow area = in-season, Green area = mid-season winter break.

The mean weekly prevalence of health problems, injuries and illnesses were 20.1%, 16.5% and 3.8%, respectively. Sudden-onset and gradual-onset injuries accounted for 34.8% and 47.8% of the total injury prevalence. Mean weekly prevalence of all health problems, as well as health problems of substantial and time-loss character, is reported in Table 4. During the study period, fluctuations in health problem, injury and illness prevalence were observed (Figure 3).

**Table 4:** Weekly health problem prevalence in danish male elite youth football players (*N* = 223). Values are presented as mean percentages with 95% confidence intervals (CI). .

	Mean (95% CI)	
All health problems	20.13%	(18.50–21.76%)
Injuries	16.48%	(15.28–17.68%)
Sudden-onset injuries	7.01%	(6.38–7.64%)
Gradual-onset injuries	9.63%	(8.70–10.55%)
Illness	3.83%	(2.82–4.83%)
Substantial health problems	13.65%	(12.45–14.85%)
Injuries	10.89%	(10.04–11.73%)
Sudden-onset injuries	5.13%	(4.57–5.70%)
Gradual-onset injuries	5.80%	(5.13–6.47%)
Illness	2.91%	(2.06–3.77%)
Time-loss health problems	6.59%	(5.88–7.29%)
Injuries	5.58%	(5.01–6.16%)
Sudden-onset injuries	3.07%	(2.64–3.51%)
Gradual-onset injuries	2.53%	(2.08–2.98%)
Illness	1.05%	(0.58–1.53%)

### Injury incidence and injury burden

**Figure 4:**
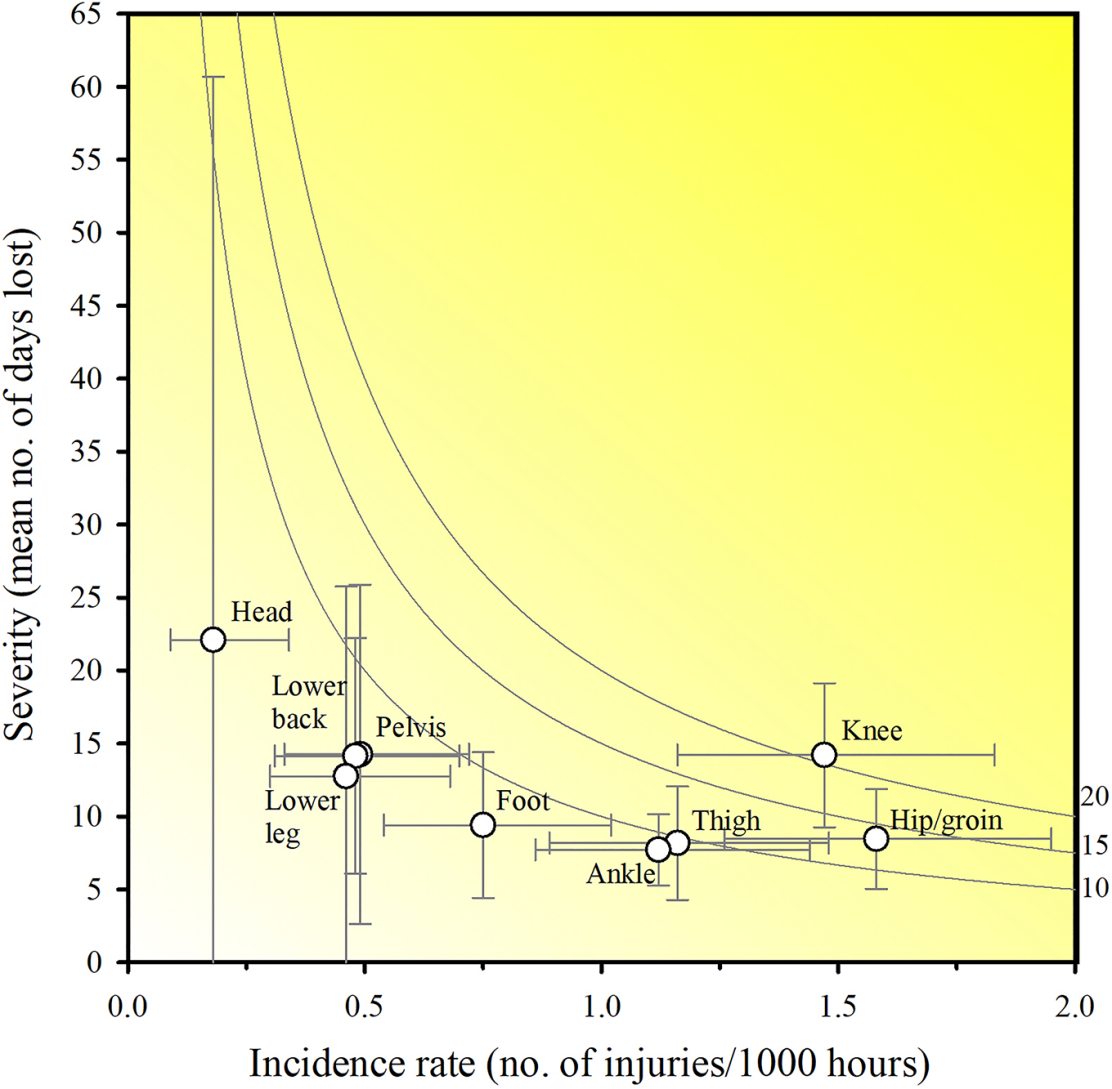
Risk matrix based on Oslo Sports Trauma Research Center questionnaire on health problems. Mean time-loss days illustrating the burden of injuries. Error bars represent 95% CIs. Incidence calculations are based on the total football exposure. A darker shade represents a greater burden with isobars indicating equal burden lines.

**Figure 5:**
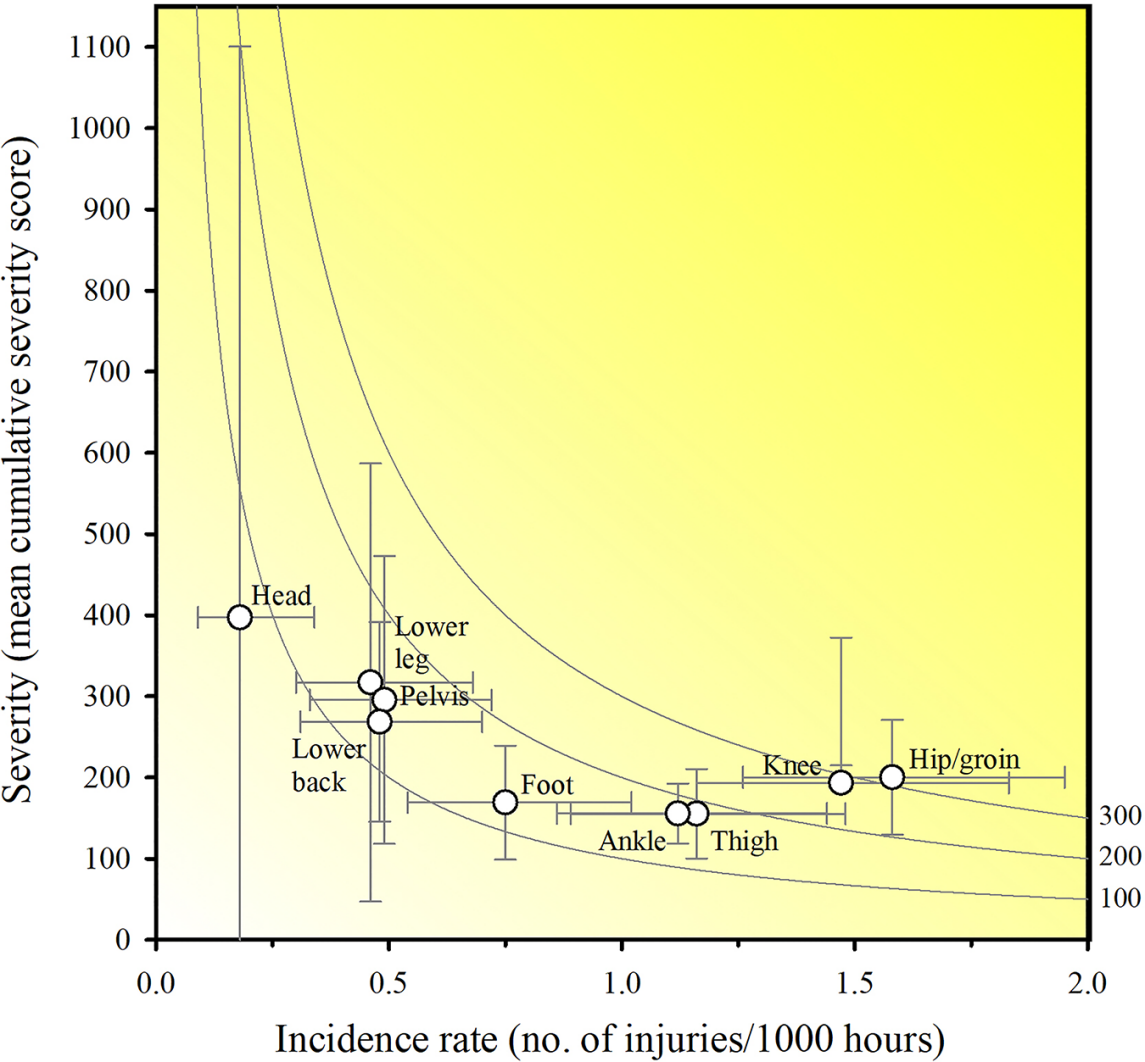
Risk matrix based on Oslo Sports Trauma Research Center questionnaire on health problems. Mean cumulative severity scores illustrating the burden of injuries. Error bars represent 95% CIs. Incidence calculations are based on the total football exposure. A darker shade represents a greater burden with isobars indicating equal burden lines.

The injury incidence rate was 8.28 (95% CI: 7.54 to 9.08) per 1,000 h of football exposure, with injury incidence rate for team training and match play being 7.24 (95% CI: 6.50 to 8.04) and 16.77 (95% CI: 13.65 to 20.4) per 1,000 h of football exposure, respectively, resulting in a match play to team training incidence rate ratio of 2.3. All injuries sustained during matches (*N* = 100) were categorized as having a sudden-onset, whereas the injury incidence in training for sudden-onset and gradual-onset was 2.23 (95% CI: 1.83 to 2.69) and 5.01 (95% CI: 4.4 to 5.68) per 1,000 h of exposure, respectively. Hip/groin injuries had the highest incidence rate of 1.58 (95% CI: 1.26 to 1.95) per 1,000 h of football exposure, while knee injuries had the highest injury burden with 20.86 days lost per 1,000 h of football exposure. Elbow and head injuries resulted in the highest mean time-loss days of all body regions, with a mean of 35.0 ± 0.0 (*N* = 1, hence no SD) and 22.1 ± 53.95 days lost per injury, respectively. Injury incidence and burden are presented in Table 5. Risk matrixes displaying the overall incidence and severity of injuries for each body region is presented in Figures 4 and 5.

**Table 5:** Injury number, Incidence rate, time-loss, and burden per body region. Total and mean values related to injury incidence rates are presented with 95% confidence intervals (CI). Values related to the specific body regions and total values are presented as absolute values. Total and mean values related to time-loss days are presented with mean (SD).

Body region	No. of injuries	Incidence rate, injuries / 1,000 h	Time-loss days	Burden, Time-loss days / 1,000 h
Head	10	0.18 (0.09 to 0.34)	22.1 (53.95)	4.06
Neck	3	0.05 (0.01 to 0.16)	3.33 (2.52)	0.18
Shoulder	4	0.07 (0.02 to 0.19)	1.0 (2)	0.07
Elbow	1	0.02 (0.0 to 0.1)	35.0 (0)	0.64
Forearm	1	0.02 (0.0 to 0.1)	0.0 (0)	0.0
Wrist and hand	13	0.24 (0.12 to 0.41)	7.77 (8.98)	1.85
Chest and upper back	2	0.04 (0.0 to 0.13)	1.5 (2.12)	0.05
Abdomen	1	0.02 (0.0 to 0.1)	9.0 (0)	0.16
Lower back	26	0.48 (0.31 to 0.7)	14.15 (19.94)	6.76
Pelvis	27	0.49 (0.33 to 0.72)	14.26 (29.4)	7.07
Hip/groin	86	1.58 (1.26 to 1.95)	8.46 (15.92)	13.37
Thigh	63	1.16 (0.89 to 1.48)	8.17 (15.47)	9.46
Knee	80	1.47 (1.16 to 1.83)	14.2 (22.22)	20.86
Lower leg	25	0.46 (0.3 to 0.68)	12.76 (31.49)	5.86
Ankle	61	1.12 (0.86 to 1.44)	7.72 (9.51)	8.65
Foot	41	0.75 (0.54 to 1.02)	9.41 (15.84)	7.09
Unspecified	7	0.13 (0.05 to 0.26)	2.95 (7.8)	0.88
Total	451	8.28 (7.54 to 9.08)	10.69 (18.5)	87.04

### Player availability

**Figure 6:**
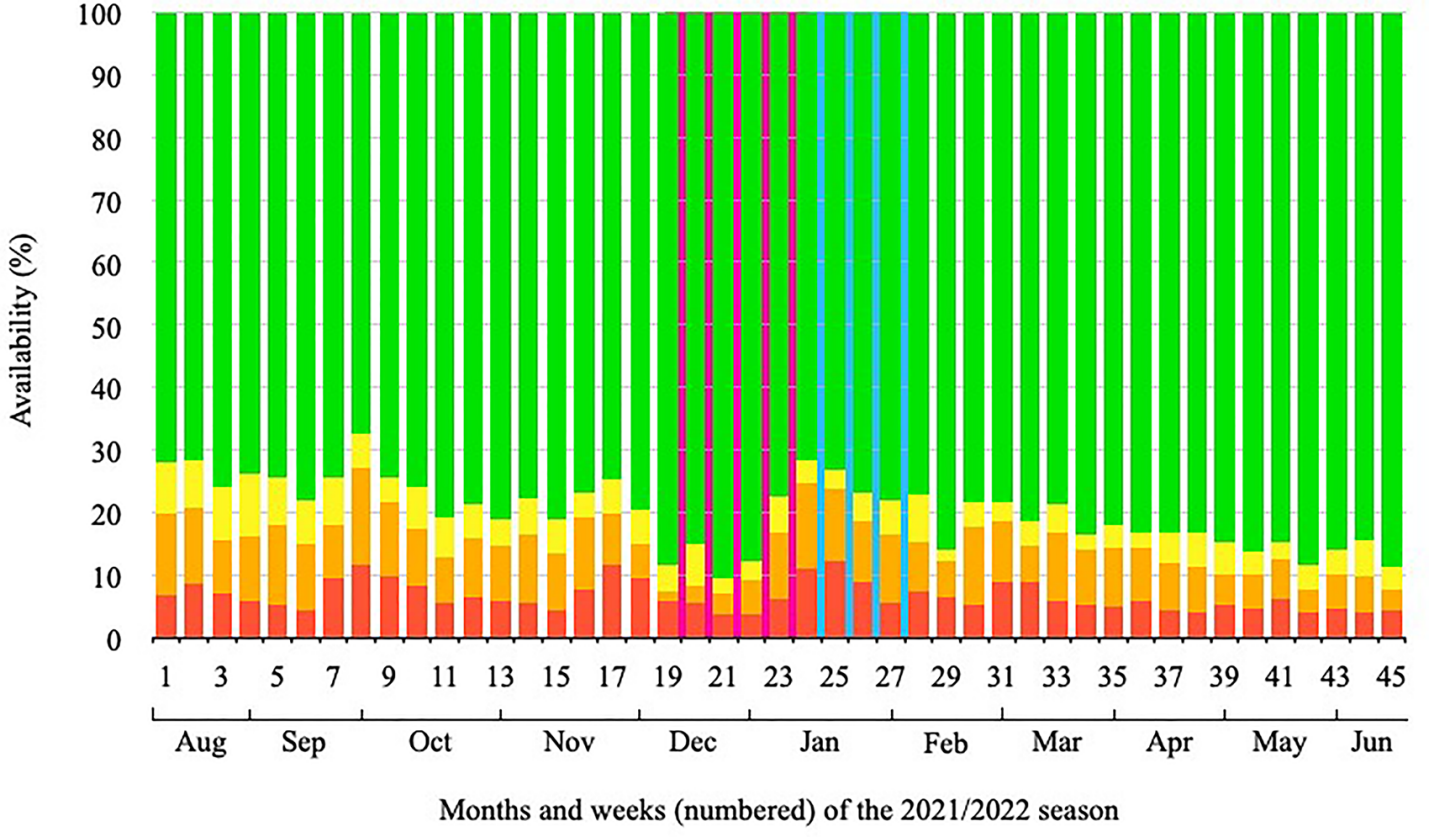
Weekly player availability (%) during the 45-week study period. Green area = full participation, yellow area = full participation but with a health problem, orange area = reduced participation due to a health problem, red area = no participation due to a health problem.

The mean weekly proportion of players able to fully participate in football with or without a health problem during the study period was 84.9% (95% CI: 83.5 to 86.3). The average weekly proportion of players who were able to participate fully but had a health problem was 5.04% (95% CI: 4.48 to 5.6). During the study period the mean weekly proportion of players fully unable to participate in any football activities was 6.6% (95% CI: 5.9 to 7.3). On average, the number of weeks a player was unable to participate in any football activities was 2.7 (95% CI: 2.12 to 3.24) during the study period. The player availability during the full study period is presented in Figure 6.

## Discussion

During a full playing season, our study prospectively investigated health problems in Danish male youth elite football players. A major finding is, that the injury pattern of young elite football players below the age of 17 years resembled that of adult level professional players (6). As such, lower extremity sudden-onset and gradual-onset injuries was shown to be the main causes of missed team practice and match play. Furthermore, knee and head injuries displayed the highest burden and had the most days lost per injury, respectively. Also, a higher injury incidence rate per 1,000 h was observed during match play compared to team practice. Another finding of the study is, that the weekly prevalence of health problems fluctuated over the time-course of the study. This indicates that injury risk in youth footballers may be different at various stages during a playing season. In addition, the current study reveals, that young football players take part of a significant number of weekly supportive activities (i.e., strength training, self-practice etc.). This finding suggests that football exposure *per se*, may not reflect the actual physical load of contemporary youth elite football.

The total injury incidence rate in elite youth football players is in our study 8.3 injuries per 1,000 h of football exposure. This is higher compared to estimates (6.19 injuries/1,000 h) presented in a recent meta-analysis (23), and lower than reported for similar age-group players from the Netherlands (10.1 injuries/1,000 h) and Qatar (17.0 injuries/1,000 h (18, 24). These discrepancies may be related to differences in study designs, clubs, age-groups, and to individual training and match exposure. In our study, a broader definition of health problems was applied. This is suggested to potentially reveal more health problems (e.g., minor injuries, mild illness symptoms, and mental health issues) compared to other methods (19). Furthermore, the current investigation was conducted across several clubs, each applying a unique approach to player development (25). In addition, the average weekly football exposure in the present study was comparable to that recently demonstrated in under-19 international level players (26). Lastly, the players weekly conducted several hours of non-coach lead practice (Table 2). Collectively, this may influence our results compared to other studies.

The burden of injuries during the study period is 87 time-loss days per 1,000 h of football exposure (Table 5). This is low when compared to well-trained youth players (425 and 316 injury days/1,000 h of football exposure for U16 and U17 male elite football players, respectively) (18). It is reasonable to suggest, that youth elite footballers generally have access to necessary resources such as equipment, comprehensive medical support, and expert coaches with the ability to control match and training load. Therefore, the reason for this discrepancy is not clear. However, several teams recruited for the present investigation competed at international level standards. As such, it is possible that we have included players who are more experienced, stronger, physically fit, and thus more resistant to injuries.

In this study, gradual-onset injuries account for 43.4% of the total proportion and 32.**7**% of all health problems, respectively. In youth football, between 10 and 40% of all injuries are reported to be the result of gradual-onset (27), with differences in definitions being a major contributor to this considerable variation. Our findings are comparable to that in adult elite players, whereas a markedly higher match injury incidence rate is consistently reported for adults (28). It is an unexpected finding in our study, that all match injuries are reported as being of sudden-onset nature. As such, difficulties in assessing the underlying cause behind an injury may influence our results (29). Alternatively, players with pre-match clinical signs of over-use may have been excluded from match participation after medical examination. Also, the types of gradual-onset injuries appear to be age-dependent, with tendinopathies and growth-related conditions being more prevalent in young players. For example, Osgood-Schlatter disease is a common major injury peaking in the under-13 and under-14 age groups, whereas Sever's disease is most frequent in the under-11 age group (30). Although previously established (18), sudden-onset injuries may thus not always differentiate under-17 youth and adult elite football players (5). Finally, a significant proportion (23,6%) of the players were either excluded or left the study, with the remaining players upholding a high response rate throughout the study period. As such, selection bias is likely to have influenced our findings.

In our study, a significant proportion of all reported health problems are related to injuries in the lower extremity. This is in alignment with previous reports on youth elite football (5, 31, 32). The physical demands of elite youth football include high-intensity runs, frequent tackling situations and exposure to collisions and contacts (33). This is believed to increase lower extremity injury risk (27). Also, we report a higher match injury and sudden-onset injury incidence per 1,000 h of exposure compared to team practice. A more pronounced accumulation of physical fatigue as well as an increased number of contacts and collision may influence injury incidence rates during matches (34). Furthermore, a decreased predictability of specific game situations when facing opposing teams may substantially increase match play injury risk (35).

Knee injuries display the highest burden in this study. Knee injuries represent 17% (7%–23%) of all injuries in male youth players (32). In addition, one-third are reported to be due to poor knee function and to occur without contact (36, 37). It has previously been shown that 10–15 min of neuromuscular training 2–3 times weekly reduces non-contact injuries by 45% in youth football players (38). However, although the provision and application of injury treatment and rehabilitation services appears to be adequate in elite football, the provision of injury prevention services is not (39). Therefore, medical and sports science staff may benefit from revising their injury prevention strategies and placing more emphasis on the most burdensome injuries.

Elbow and head injuries have the most time-loss days per injury in present study. Head injury incidence rate in football (soccer) is commonly reported to be low (40) and comparable to our current findings (10 injuries; 0.1 injuries/1,000 h). However, head injuries may substantially impair a youth player's ability to perform at the elite level. Head injuries may also be subjected to inconsistencies in the interpretation and reporting of the symptoms and are hence frequently underdiagnosed (41). As such, future prospective studies using accurate definitions, recognition, and report on this type of injury are needed to comprehensively study the incidence of concussions among youth footballers. In this study, a single elbow injury (due to a fracture) sustained during a match was reported (Table 5). Fractures represent 4% of all injuries in elite football, with 23% of traumatic football fractures occurring in the upper extremity in professional players. Match-play related fracture incidence rate has been reported to be 12-times higher than training fracture incidence rate. This may be explained by differences in playing intensities between training and matches (42). Mean time to return to football after a proximal forearm fracture has been reported to be 5.3 wks (43). As such, fractures, despite their relatively low representation, denote one of the most serious injuries incurred by football players, and account for the most time to recover post-injury. This is confirmed by our findings.

Visual inspections reveal marked fluctuations in health problem, injury, and illness prevalence (Figure 3) during the study period with concomitant variability in player availability (Figure 5). Also, health problems, injury, and illness prevalence appear to decrease during the latter part of the study period compared to the former. Several factors may explain these findings. Our study included young players from elite football academies. During their academy training, players undergo several phases of transitioning from one playing level or team to the next. These transitions usually occur after the summer break and are often characterized by changes in coaching and support staff, squad composition, training processes, and playing intensities. In fact, football players may not always acquire the necessary levels of fitness during preseason conditioning to resist the load associated with playing competitive football (4). Consequently, the above may have influenced the risk of illnesses as well as of sudden-onset and gradual-onset injury at the beginning of the study period (32). Throughout the duration of the study period, the players dedicated a substantial amount of time to strength/power training and injury prevention activities (Table 2). It is thus reasonable to speculate, that the players may have adapted to the level and intensity of football exposure and reduced their risk of injury. Alternatively, the coaching staff may have adjusted the training processes to better align with the players' capacities as the players were exposed to a significant amount of football activities on a weekly basis. Lastly, it is important to acknowledge that the decline observed may potentially be attributed to player responder fatigue.

Using time-loss as a single measure of injury severity to assess health problem burden, may underrepresent gradual-onset injuries and fail to address the most important injuries (19). Our study presents risk matrices to identify the importance of each football-related injury (Figures 4 and 6). This approach shows injuries located to the knee to be the most burdensome in our study. Knee injuries alongside ankle, thigh and hip/groin injuries have consistently been reported to have a high prevalence in football (23). In youth football, lower extremities injuries represent about four out of five injuries with thigh, ankle, knee, and hip/groin injuries accounting for 25% (range: 11%–39%), 18% (range: 9%–31%), knee 17% (range: 7%–23%), and 14% (2%–33%) of all injuries, respectively (32). However, isolated proportions for body parts and types are of limited value since they do not consider injury severity. Reporting injury burden (i.e., days lost for combinations injury types) may therefore represent an advancement in our understanding (44). For instance, in a study of male academy football players, thigh muscle injuries were the most frequent (16% of all injuries, accounting for 11% of total time-loss), whereas joint sprains to the knee had the greatest impact on player availability (3% of injuries and 18% of total time-loss) (18). This information is lost if severity is not accounted for. Therefore, using more measures of health problem severity may provide additional insight, and the information could potentially help practitioners improve the injury-prevention measures used.

Weekly throughout the study period, on average 84.91% (95% CI: 83.5 to 86.32) of all players were able to fully participate in football without or with only a minor health problem. Hence, the player availability in this study is comparable to previous findings reporting an overall player availability of 82.7% in youth academy players (18). Assuming a team size of 20 players, 17 players should at any given time point during the season be available for full participation in practice or matches. In addition, two players should be expected to participate at a reduced level, whereas one player should not be able to participate due to an injury or illness. Being able to participate (i.e., having more football exposure), is suggested as a factor for development success in professional football (45). As presented in this study, a youth elite football player must expect to be unable to participate for on average 2.7 weeks per season. Extending this, although injury incidence rates and time-loss may vary greatly, approximately 4 months of health problem related time-loss must be accounted for during a six year-long academy career. Being away from football for more than 28 days due to a severe injury has been suggested to affect playing skills, long-term development, health outcomes, and future career opportunities significantly (4, 46). As such, introducing effective measure to secure a safe and timely return to football after an injury should be a main priority for football academy medical and sports science practitioneers to optimize player development.

This study took place during the later stages of the COVID-19 pandemic, with the prevalence of COVID-19 remaining at a low level throughout the study period. Also, it is worth mentioning that nearly 80% of all COVID-19 positive tests were reported immediately after the mid-season winter break. Therefore, the impact of COVID-19 on our results may be considered negligible, although the long-term relationship between COVID-19 and changes in injury risks among elite football players is only sparsely investigated (47).

Several limitations apply to our study. For instance, our study includes self-reported football exposure which may introduce measurement errors. However, collecting data through athlete management systems (questionnaire collection) may actually facilitates more accurate measures of exposure (48). In addition, this study does not report categories of tissue or injury pathology as recommended by others (19). From a practical and a research perspective, it is relevant to obtain as much information as possible about an injury, and this would have qualified our findings further. Also, the study includes participants from a selected group of male elite level football players below the age of 17 years. Injury risk in football may depend on gender, playing level and age-group. Therefore, extending our findings to other groups of players or other high-risk team sports should be done with caution. Finally, specific playing positions (e.g., goal keepers) may be subjected to different injury risk profiles (49). However, this is less well-described in youth elite football players (17) and is subject to future investigations.

In summary, during the investigation period, the main causes of missed team practice and match play in young male elite football players were lower extremity sudden-onset and gradual-onset. Injury incidence per 1,000 h was higher during match play compared to team practice, and the players took part of a significant number of weekly supportive activities. The injury pattern in youth players was comparable to that of adult players, with knee and head injuries displaying the highest burden and resulted in the most days lost per injury, respectively. As such, effective monitoring of health problems and physical exposure may guide medical and sports science practitioneers to optimize training and recovery processes, and to secure player development and long-term academy productivity.

## Practical recommendations

Implementing a continuous monitoring system across age groups and teams will allow for the identification of players at particular risk during transition phases or other periods of increased risk (e.g., after holiday periods, national team camp breaks, or during congested match fixtures). Applying this approach may aid sports science staff members in adapting their training processes to align with the players' capacities at different time points throughout the season.

In this study, a web application was used to collect player data, and a high response rate was achieved. To ensure accurate and timely responses to the injury survey, it is advisable to assign dedicated staff members with the responsibility of guiding the players, as demonstrated in this study. This may increase the validity of the collected data and facilitate subsequent data processing and analysis. Similarly, it is advised to gather sufficient information on injury mechanisms regardless of the type of injury and to include injury burden as a tool for analysis. This will improve the possibility of tailoring individual injury prevention and performance-enhancing training processes. Including options for reporting self-initiated training activities may be beneficial, as not all training forms are included in load management models presently applied in elite football. This inclusion has the potential to enhance the identification of players at risk of sustaining undesirable states of overtraining and thus decrease the risk of gradual-onset injuries.

## Data Availability

The raw data supporting the conclusions of this article will be made available by the authors, without undue reservation.
